# Association Between Verbal Communication With Families and Delirium in Mechanically Ventilated Patients: A Retrospective Study

**DOI:** 10.7759/cureus.73513

**Published:** 2024-11-12

**Authors:** Kousei Kudou, Kazuki Okura, Kasumi Satoh

**Affiliations:** 1 Department of Nursing, Akita University Hospital, Akita, JPN; 2 Department of Rehabilitation Medicine, Akita University Hospital, Akita, JPN; 3 Department of Emergency and Critical Care Medicine, Akita University Graduate School of Medicine, Akita, JPN

**Keywords:** communication, critical care, critical care nursing, delirium, family, intensive care unit, intermittent positive-pressure ventilation

## Abstract

Introduction

Delirium in the intensive care unit (ICU) significantly impacts patient outcomes. While family involvement may help prevent delirium in ICU patients, its effect during mechanical ventilation remains unclear. This study investigated the association between verbal communication during family visits and early post-extubation delirium in mechanically ventilated ICU patients.

Materials and methods

This retrospective, analytical observational study used data from the hospital's standard electronic health record (EHR) system, which includes routine medical and nursing documentation. We conducted this study in a 10-bed emergency ICU at an academic hospital in rural Japan from April 1, 2019, to December 31, 2020. We included patients aged ≥18 years who received invasive positive pressure ventilation for >48 hours. We excluded patients who were discharged while intubated, required a tracheostomy, or were unable to be assessed using the intensive care delirium screening checklist (ICDSC). The primary outcome was the maximum ICDSC score within 24 hours post-extubation. We conducted multiple regression analyses with ICDSC as the outcome.

Results

A total of 110 patients were analyzed. The mean age was 64.7 years (standard deviation ± 14.3), and 73 patients were male (66.4%). The median duration of intubation was five days (interquartile range (IQR), 4-8), and the median sequential organ failure assessment (SOFA) score was 8 (IQR, 6-11). The median ICDSC within 24 hours post-extubation was 3 (IQR, 2-5). Of the total 110 patients, 28 (25.5%) could communicate verbally. Patients who could communicate verbally had lower SOFA scores, longer duration of intubation, more frequent family visits, and higher intensive care mobility scale (IMS) scores during intubation. Neither single nor multiple regression showed a significant association between verbal communication and post-extubation ICDSC.

Conclusions

We did not find a statistically significant association between verbal communication during family visits with patients and ICDSC within 24 hours post-extubation. Potential confounders and variations in care practices may have influenced the results. Further studies are needed to address these limitations.

## Introduction

In recent years, there has been an increasing emphasis on the participation of the patient's family in visiting and caring for the patient in the intensive care unit (ICU) [1]. The primary goal of ICU care is to rapidly resolve life-threatening conditions through multidisciplinary treatment [2]. While addressing immediate life-threatening conditions is paramount, it is also important to consider the long-term implications, particularly post-intensive care syndrome (PICS). PICS refers to physical, cognitive, and psychiatric impairments occurring in patients who have experienced intensive care [3], and numerous reports have shown that patients’ quality of life is impaired long after they leave the ICU [4,5].

The 'ABCDEFGH' bundle is a comprehensive care strategy for ICU patients, incorporating various elements to enhance outcomes [6]. This approach recognizes 'family involvement' as a crucial component in PICS prevention, acknowledging families as both care recipients and potential caregivers for critically ill patients. The quality of family involvement plays a significant role in PICS prevention and patient outcomes.

Furthermore, this integrated care model includes 'delirium monitoring and management' as key aspects of patient care [6]. Delirium has a significant impact on ICU patients, worsening outcomes. It is also interrelated with pain, agitation, immobility, and sleep disturbances [7]. Importantly, delirium is not only an acute concern but also a significant risk factor for developing long-term cognitive impairments associated with PICS. Given these adverse effects of delirium and its potential contribution to PICS, researchers have explored various prevention strategies, including family involvement.

A systematic review by Chen et al. suggests that family involvement reduces the incidence of ICU delirium [8], and several other studies have indicated that family involvement is essential for delirium prevention [9-12]. While the importance of family involvement is clear, the optimal methods of involvement require further investigation. Merely extending the duration of family visits does not reduce the incidence of delirium [13]. Therefore, evaluating the quality of communication with family members is critical. It has been reported that family members who have received appropriate communication education about delirium have reduced delirium symptoms when in contact with the patient [14]. In addition, Sahawneh and Boss reported that multifaceted interventions, including family participation, provision of familiar items, patient re-orientation, and increased family visitation time, were associated with reduced occurrence and duration of delirium [15]. Based on these findings, we consider it essential to focus on both the quantity (frequency and duration) and quality of interactions during family visits to effectively prevent delirium and potentially mitigate PICS.

However, most studies on family visits and delirium have focused on the post-extubation period, while little attention has been given to the potential impact of family interaction during mechanical ventilation. Mechanical ventilation is a life-supporting treatment that assists or replaces spontaneous breathing in patients with conditions such as respiratory failure, neurologic disorders, or severe injuries. This is achieved by delivering oxygenated air into the lungs through an endotracheal tube connected to a ventilator machine [16]. This lack of research on family interaction during mechanical ventilation represents a significant gap in the literature. Mechanically ventilated (MV) patients, when appropriately managed with analgesia and sedation, can maintain a level of consciousness that allows interaction. They can communicate their thoughts using tools such as pen and paper or communication boards.

Given the complexity of family involvement during mechanical ventilation, we focused on two measurable aspects: the frequency of family visits and the presence of verbal communication. This study aimed to investigate the association between verbal communication with families and delirium in MV patients. We examined whether patients who could engage in verbal communication during family visits showed different delirium outcomes compared to those who could not. Our findings may contribute to developing evidence-based strategies for delirium prevention and inform ICU protocols on family involvement during mechanical ventilation.

## Materials and methods

Study design and ethics 

This retrospective, analytical observational study used data from the hospital's standard electronic health record (EHR) system, which includes routine medical and nursing documentation. The study design and data analysis were conducted in accordance with the principles of the Declaration of Helsinki and ethical guidelines for medical research in Japan.

This study analyzed data retrieved from the EHRs of a 10-bed emergency ICU at an academic hospital in rural Japan. The ICU was semi-closed, with a nurse-to-patient ratio of 1:2. The period under analysis was from April 1, 2019, to December 31, 2020.

The Akita University Graduate School of Medicine Ethics Committee approved this study in March 2023 (approval no. 2951). The Ethics Committee waived the requirement for informed consent, due to the study's observational nature and the absence of treatments beyond routine clinical practice.

Measurements

Verbal Communication

Verbal communication was operationally defined as the patient's ability to convey meaningful messages in response to family members' verbal cues, using written means such as pen-and-paper or communication boards. This included writing at least one contextually appropriate word or using symbols to express clear ideas or responses. We did not distinguish between single-word responses and full sentences, as both were considered indicative of the patient's ability to engage in meaningful communication. This occurred during successful spontaneous awakening trials [6] when patients were sufficiently alert and capable of interaction.

Family Visits

Family visits were counted when the patient's family came to the bedside. During these visits, families typically engaged in various activities, such as looking at the patient, touching the patient, talking to the patient, and engaging in bidirectional communication (e.g., the patient responding to family members' verbal or non-verbal cues through eye movements, gestures, or written responses). The frequency of visits was recorded in the nursing documentation.

Data collection 

This retrospective observational study analyzed data from ICU admissions during the study period (April 1, 2019, to December 31, 2020) that met the following inclusion criteria: age 18 years or older at ICU admission, and receipt of invasive positive pressure ventilation in the ICU for more than 48 hours. Cases were excluded if they documented ICU discharge while still intubated, the requirement for tracheostomy due to inability to be extubated, or inability to be assessed using the intensive care delirium screening checklist (ICDSC). As this study was a retrospective analysis of all eligible cases within the specified timeframe, no specific sampling method was applied.

We collected data on our primary variables of interest (number of family visits and presence of verbal communication) and known risk factors for delirium. The risk factors included gender, age, sequential organ failure assessment (SOFA) score at ICU admission, duration of intubation, intensive care mobility scale (IMS) scores during and after intubation, and neurological dysfunction (impaired consciousness, cerebral neurological diseases, and dementia). The SOFA score assesses six organ systems (respiratory, cardiovascular, hepatic, coagulation, renal, and neurological) on a scale of 0-4 each, with higher scores indicating more severe dysfunction [17]. The IMS is an 11-point scale (0-10) measuring patient mobility in the ICU, ranging from lying in bed (0) to walking independently without a gait aid (10), and has shown excellent inter-rater reliability [18]. IMS was assessed by bedside nurses every 12 hours (at each shift), recording the maximum value. We used the ICDSC to assess delirium. The ICDSC is an eight-item screening tool that evaluates key delirium symptoms, with a total score of 4 or greater indicating the presence of delirium [19]. The ICDSC was evaluated by bedside nurses at least every six hours daily, recording the maximum value.

The primary outcome was the maximum ICDSC score within 24 hours post-extubation. Details of family visits and verbal communication, including written responses from MV patients, were extracted from nursing records, while other patient attributes and treatment information were obtained from medical records. As nurses worked two shifts daily, intubation duration was counted in 0.5-day increments.

Data analysis

We presented continuous variables as mean ± standard deviation or median (first quartile to third quartile) based on their distribution, and categorical variables as frequencies and percentages. Between-group comparisons (verbal communication vs. no verbal communication) were conducted using t-tests for normally distributed continuous variables, Mann-Whitney U tests for non-normally distributed continuous variables, and Fisher's exact tests for categorical variables. ICDSC and IMS scores were treated as continuous variables.

A propensity score adjustment [20] was performed to account for potential confounding factors. The propensity score was calculated using logistic regression with variables that could potentially influence both the ability to communicate verbally and the presence of delirium during mechanical ventilation, including the SOFA score, age, gender, presence of neurological dysfunction, and IMS score during intubation. The propensity score was then included as a covariate in the multiple regression analysis to adjust for potential confounding. The independent variables for the multiple regression analysis were deliberately selected based on prior studies, available data in the electronic medical records, and the researchers' clinical experience. These variables included duration of intubation, verbal communication, number of family visits, surgery, IMS within 24 hours post-extubation, and the propensity score. ICDSC was set as the dependent variable. The regression analysis results were presented with coefficients and their corresponding 95% confidence intervals (95% CIs).

All statistical tests were two-tailed, and a p-value <0.05 was considered statistically significant. Data analysis was performed using R software for Microsoft Windows, version 4.3.1 (R Foundation for Statistical Computing, Vienna, Austria).

## Results

A review of EHRs identified 209 patients who met the inclusion criteria during the study period. After applying exclusion criteria, data from 110 patients were analyzed. Table 1 provides an overview of the 110 MV patients in the study. The mean age was 64.7 (standard deviation ± 14.3), and 73 patients were male (66.4%). The median duration of intubation was five (interquartile range (IQR), 4-8) days, and the median SOFA score was 8 (IQR, 6-11). In terms of surgery, 41 patients (37.3%) underwent surgery, and 25 patients (22.7%) had neurological dysfunction before intubation. The median ICDSC within 24 hours post-extubation was 3 (IQR, 2-5). The median IMS score during intubation was 1 (IQR, 1-1), and the median IMS score within 24 hours post-extubation was 1 (IQR, 1-3). The median number of family visits was 1 (IQR, 1-3). 

**Table 1:** Characteristics of the participants SOFA: sequential organ failure assessment; IMS: intensive care unit mobility scale; ICDSC: intensive care delirium screening checklist Data are presented as n (%), mean ± standard deviations, or median (first to third quartile).

Parameters	All participants (n = 110)
Age	64.7 ± 14.3
SOFA score	8 (6-11)
Duration of intubation	5 (4-8)
Number of family visits during intubation	1 (1-3)
IMS during intubation	1 (1-1)
IMS within 24 hours post-extubation	1 (1-3)
ICDSC within 24 hours post-extubation	3 (2-5)
Gender, male	73 (66.4%)
Surgery	41 (37.3%)
Neurological dysfunction	25 (22.7%)

Table 2 provides a comparison of the verbal communication and non-verbal communication groups. Based on nursing documentation, 28 patients (25.5%) had recorded instances of verbal communication with family during intubation. The group of MV patients who were able to communicate verbally had lower SOFA scores (p < 0.05), longer duration of intubation (p < 0.01), more frequent family visits (p < 0.01), and higher IMS during intubation (p < 0.05).

**Table 2:** Comparison of verbal communication and non-verbal communication groups SOFA: sequential organ failure assessment; IMS: intensive care unit mobility scale; ICDSC: intensive care delirium screening checklist Data are presented as n (%), mean ± standard deviations, or median (first to third quartile). Statistical significance was determined using the t-test, Mann-Whitney test, or Fisher's exact test comparing verbal communication vs. non-verbal communication group. *p < 0.05; **p < 0.01

Parameters	Verbal communication (n = 28)	Non-verbal communication (n = 82)	p-value
Age	60 ± 17	67 ± 13	0.06
SOFA score*	7 (5-9)	9 (6-11)	0.04
Duration of intubation**	8 (5-13)	5 (3-7)	<0.01
Number of family visits during intubation**	4 (2-7)	1 (0-2)	<0.01
IMS during intubation*	1 (1-3)	1 (1-1)	0.01
IMS within 24 hours post-extubation	1 (1-3)	1 (1-3)	0.58
ICDSC within 24 hours post-extubation	3 (1-5)	3 (2-4)	0.64
Gender, male	16 (57.1)	57 (69.5)	0.25
Surgery	10 (35.7)	31 (37.8)	1.00
Neurological dysfunction	8 (28.6)	17 (20.7)	0.44

Table 3 provides the results of single regression analysis. The single regression analysis showed no significant association between verbal communication during mechanical ventilation and ICDSC within 24 hours post-extubation.

**Table 3:** Association between verbal communication and delirium using single regression analysis β: coefficient; SE: standard error; 95% CI: 95% confidence interval Statistical significance was determined using single regression analysis.

Variables	β	SE	95% CI	p-value
Verbal communication during intubation	-0.19	0.45	-1.10, 0.71	0.67

Multiple regression analysis results are presented in Table 4. Multiple regression analysis with ICDSC within 24 hours post-extubation as the dependent variable showed no significant association with surgery (β = -0.07, 95% CI: -0.89 to 0.75, p = 0.87), duration of intubation (β = -0.03, 95% CI: -0.14 to 0.09, p = 0.66), number of family visits during intubation (β = 0.15, 95% CI: -0.07 to 0.37, p = 0.19), verbal communication during intubation (β = -0.34, 95% CI: -1.49 to 0.81, p = 0.56), and IMS within 24 hours after extubation (β = -0.21, 95% CI: -0.53 to 0.10, p = 0.18).

**Table 4:** Association between verbal communication and delirium using multiple regression analysis β: coefficient; SE: standard error; 95% CI: 95% confidence interval; IMS: intensive care unit mobility scale Statistical significance was determined using multiple regression analysis.

Variables	β	SE	95% CI	p-value
Surgery	-0.07	0.42	-0.89, 0.75	0.87
Duration of intubation	-0.03	0.06	-0.14, 0.09	0.66
Number of family visits during intubation	0.15	0.11	-0.07, 0.37	0.19
Verbal communication during intubation	-0.34	0.58	-1.49, 0.81	0.56
IMS within 24 hours post-extubation	-0.21	0.16	-0.53, 0.10	0.18

## Discussion

This retrospective analysis of EHR data found no significant association between the ability of MV patients to verbally communicate with their families and delirium symptoms within 24 hours post-extubation. While previous studies [9-12] have generally demonstrated that family visits and high-quality communication reduce delirium in critically ill patients, our study presents contrasting results, which may be partially explained by our focus solely on verbal communication. Previous studies have shown that family visits can alleviate delirium, but also anxiety [21], confusion, and agitation [22]. These factors may interact, potentially creating a cascading effect on delirium reduction. Moreover, family visits have various beneficial effects: family members provide emotional support and often participate in care [23]. Their presence and involvement can significantly enhance patients' emotional and physical comfort [24]. Our study might not have fully accounted for these interconnected benefits of family visits by focusing solely on verbal communication. Our findings highlight the complexity of patient-family communication and suggest the need for a more comprehensive approach to preventing delirium in MV patients.

Our findings may be explained by several factors, primarily the limitations of our communication assessment. Although we defined verbal communication as meaningful exchanges, we did not assess the content or quality of these interactions. The effectiveness of communication in reducing delirium may depend on factors such as discussion topics, conversation positivity, and duration [14]. Our binary evaluation of communication ability may not adequately capture the complexity of the communication process.

Additionally, MV patients face unique challenges that may impact the effectiveness of delirium prevention strategies. Severe illness can alter a patient's cognitive and physiological state [25-27]. This may hamper the effectiveness of verbal communication in preventing delirium. Furthermore, higher doses of sedatives and analgesics, often required for MV patients, are known delirium risk factors [28] and may impair patients' ability to engage in and benefit from family interactions. Factors such as fatigue, cognitive impairment, poor coordination, and muscle weakness further complicate the communication process [29]. These barriers can lead to patient frustration and exhaustion [30], resulting in shortened or less effective family interactions.

Lastly, memory retention issues in MV patients may have significantly influenced our results. Sedatives, often administered continuously, are associated with memory impairment [31,32]. Despite efforts to reduce sedation, MV patients may require re-sedation for procedures or night rest, leading to fluctuations in consciousness levels. These factors, along with unstable sleep patterns [33], can impair memory formation and retention of family visits, potentially limiting the measurable impact on delirium symptoms.

Our findings have important implications for nursing practice. While verbal communication is crucial, nurses should not rely solely on it when developing strategies for family-patient interactions. Previous studies [34] suggest promoting an environment that encourages various forms of family engagement and support, necessitating a comprehensive approach that addresses these patients' unique challenges. This approach implies adding interventions that address memory retention and overcome communication barriers. For example, creating an ICU diary [35] with family photos and repeatedly showing it to patients could reinforce memories of family interactions that might otherwise be lost due to sedation or cognitive impairment. Additionally, enabling patients to experience written and audio messages from family members [36,37] may compensate for limitations in direct verbal communication.

Our research provides a novel perspective on family involvement in critical care by focusing on the relationship between verbal communication with MV patients and delirium symptoms. The importance of promoting early communication in MV patients, as suggested by our study, may warrant attention similar to that given to the widely promoted early rehabilitation of MV patients in recent years [38-40]. Like early rehabilitation, early communication should be an integral part of a comprehensive approach to MV patient care, addressing both the physical and cognitive aspects of recovery [41]. Our findings encourage a reconsideration of family involvement strategies in clinical settings and suggest the need for more comprehensive communication support.

Our study has several limitations. MV patients with verbal communication ability may have better cognitive and hand-motor function [25]. This could complicate the relationship we observed between communication and delirium symptoms. We addressed this by propensity score adjusting for SOFA scores and IMS in our analysis. While bedside nurses conducted delirium symptom measurements using the ICDSC, some inter-rater variability may persist despite our regular training sessions. Other potential confounders include impaired memory of family visits, which could affect the patient's ability to benefit from these interactions. Furthermore, the necessity for re-sedation in MV patients also may impact memory formation and retention, potentially affecting the patient's recollection of family visits. This adds complexity to interpreting our findings and should be considered in future studies. The lack of a formal sample size calculation is a limitation of this study. Future research should address this through appropriate pre-study power analysis, potentially informed by the effect sizes observed in our study. Our results may have been influenced by various environmental and procedural factors, including ICU operational structure, departmental variations in pain and sedation management, COVID-19-related changes in visiting policies, and admission pattern differences. As a single-center study, our results should be generalized with caution. Despite these limitations, our study reaffirms the importance of family involvement and communication in the care of MV patients.

To address these limitations and advance the field, future research should prioritize multi-center studies across diverse healthcare systems. Longitudinal studies examining the long-term effects of family involvement on patient outcomes and interventional studies testing various involvement strategies are needed. Additionally, investigating technology-mediated family involvement, especially in light of pandemic-related restrictions, would be valuable. These directions would enhance our understanding of family involvement in MV patient care and inform evidence-based practice improvements.

## Conclusions

We did not find a statistically significant association between verbal communication during family visits with MV patients and ICDSC within 24 hours post-extubation. Our focus solely on the presence of verbal communication might not have captured the full spectrum of family visits, including emotional support and care participation. The study's limitations include not assessing communication quality and potential memory retention issues in MV patients. Future research should explore comprehensive family involvement strategies, including non-verbal communication and memory reinforcement techniques, and their impact on delirium prevention in diverse critical care settings.
